# Comparative evaluation of molar distalization therapy with erupted second molar: Segmented versus Quad Pendulum appliance

**DOI:** 10.1186/s40510-014-0049-6

**Published:** 2014-08-06

**Authors:** Alberto Caprioglio, Mauro Cozzani, Mattia Fontana

**Affiliations:** Chairman Postgraduate Programme in Orthodontics, School of Medicine, University of Insubria, Varese, Italy; Professor of Orthodontics, School of Medicine, University of Cagliari, Cagliari, Italy; Research Fellow in Orthodontics, University of Insubria, Varese, Italy

**Keywords:** Class II malocclusion, Molar distalization, Non-compliance, Intraoral distalizing devices, Second molar

## Abstract

**Background:**

There are controversial opinions about the effect of erupted second molars on distalization of the first molars. Most of the distalizing devices are anchored on the first molars, without including second molars; so, differences between sequentially distalize maxillary molars (second molar followed by the first molar) or distalize second and first molars together are not clear. The aim of the study was to compare sequential versus simultaneous molar distalization therapy with erupted second molar using two different modified Pendulum appliances followed by fixed appliances.

**Methods:**

The treatment sample consisted of 35 class II malocclusion subjects, divided in two groups: group 1 consisted of 24 patients (13 males and 11 females) with a mean pre-treatment age of 12.9 years, treated with the Segmented Pendulum (SP) and fixed appliances; group 2 consisted of 11 patients (6 males and 5 females) with a mean pre-treatment age of 13.2 years, treated with the Quad Pendulum (QP) and fixed appliances. Lateral cephalograms were obtained before treatment (T1), at the end of distalization (T2), and at the end of orthodontic fixed appliance therapy (T3). A Student *t* test was used to identify significant between-group differences between T1 to T2, T2 to T3, and T1 to T3.

**Results:**

QP and SP were equally effective in distalizing maxillary molars (3.5 and 4 mm, respectively) between T1 and T2; however, the maxillary first molar showed less distal tipping (4.6° vs. 9.6°) and more extrusion (1.1 vs. 0.2 mm) in the QP group than in the SP group, as well as the vertical facial dimension, which increased more in the QP group (1.2°) than in the SP group (0.7°). At T3, the QP group maintained greater increase in lower anterior facial height and molar extrusion and decrease in overbite than the SP group.

**Conclusion:**

Quad Pendulum seems to have greater increase in vertical dimension and molar extrusion than the Segmented Pendulum.

## Background

Among several intraoral distalizing devices, the Pendulum appliance can be considered one of the most commonly used ‘non-compliance appliance’ and most effective in correcting class II molar relationship [1]. Similarly to other distalizing appliances [2-5], the Pendulum seems to correct class II molar relationship mainly by dentoalveolar changes rather than maxillary growth restriction [6]. These appliances eliminated request for compliance to the patients, but, differently to the extraoral traction, the main disadvantage can be represented by the anchorage loss, which may cause increase in treatment time and round tripping of the anchor teeth [7,8], unless reinforcement with skeletal anchorage is used [9,10].

In absence of maxillary second molar, extraoral traction and distalizing devices can be considered equally effective [11]. However, when maxillary second molar are erupted, intraoral non-compliance devices seem to be more indicated, since dentoalveolar movements can easily occur when continuous forces are applied [12].

There are controversial opinions about the effect of erupted second molars on distalization of the first molars. According to some authors [13-15], there is no significant difference in maxillary first molar movement, as well as anchorage loss, between patients who had erupted second molars and patients who did not, indicating that the eruption of second molar had minimal effect on the first molar distalization [16]. On the contrary, other authors [7,17,18] showed that the stage of eruption of the second molar may negatively influence this procedure (amount of first molar distalization, anchorage loss, and treatment duration). Karlsson and Bondemark [7] showed that distal movement of the first molar appeared to be more efficient before the eruption of the second molar, suggesting to start treatment when second molar is not yet erupted. Nevertheless, several aspects have to be yet clarified, and the presence of second molar continues to represent a much debated question: the findings reported by Antonarakis and Kiliaridis [19] are still controversial, and a recent systematic review [16] focused on distal molar movement without considering other important aspects, such as vertical dimension or anchorage loss. Moreover, in the presence of erupted second molar, it can be questionable if the maxillary molars (second molar followed by the first molar) can be sequentially distalized or if the second and first molars can be distalized together [17,18]; most of the studies did not answer this question, because distalizing devices are mostly anchored on the first molars, without including the second molars.

Therefore, the aim of the study was to compare sequential (Segmented Pendulum (SP)) and simultaneous (Quad Pendulum) molar distalization followed by fixed appliance therapy in class II patients in the presence of erupted second molar. The hypothesis was that the Segmented and Quad Pendulums showed similar dental and skeletal changes.

## Methods

**Figure 1:**
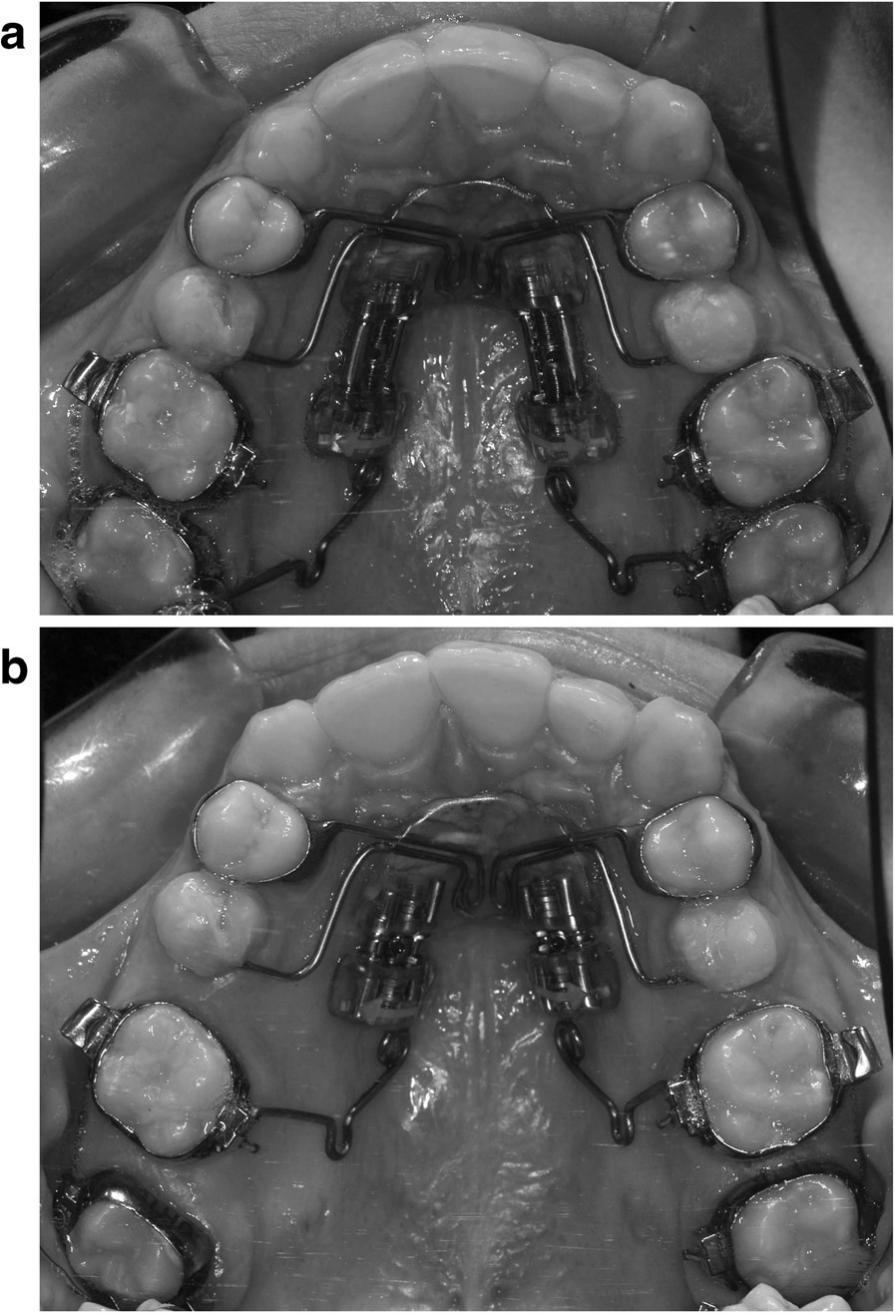
**Segmented Pendulum appliance. (a)** Before distalization and **(b)** first molar distalization.

**Figure 2:**
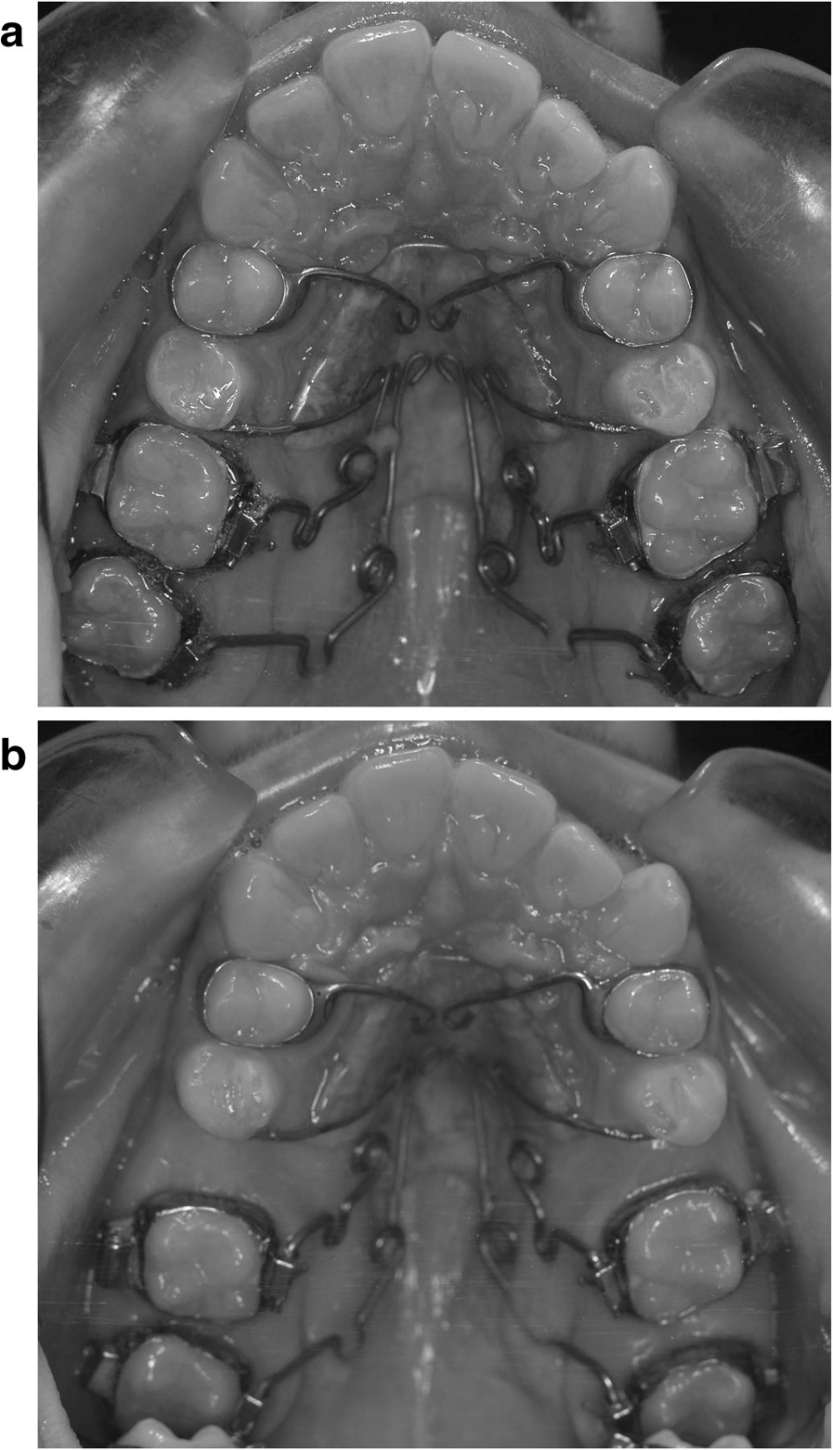
**Quad Pendulum appliance. (a)** Before distalization and **(b)** after distalization.

A sample of 44 patients was retrospectively obtained from a single orthodontic dental office. All patients were treated by a single operator and selected according to the following criteria:

– Skeletal class I or mild class II malocclusion *(A-N-Pg = 0° to 5°)* and a bilateral full cusp or end-to-end class II molar relationship

– Absence of protrusive profile or mandibular retrusion [20]

– Non-extraction treatment and absence of crowding in the mandibular arch

– Mandibular inclination (SN/Go-Gn angle) less than 37°

– Use of Segmented Pendulum (Figure 1a,b) or Quad Pendulum (Figure 2a,b) during the distalization phase

– Use of Nance button during retraction of premolar and canine

– Use of intermaxillary elastics during retraction of the maxillary incisor

– Good-quality radiographs with adequate landmark visualization and minimal or no rotation of the head

From the initial sample, seven patients were excluded from the SP group and two patients from the QP group according to the defined criteria. The records of four patients in the SP and two patients in the QP were excluded due to poor film quality or incomplete records. An additional of two patients in the SP group were excluded because the mandibular plane angle was greater than 37° and one patient because other distalizing mechanics were used between T1 and T2. Each patient was informed in detail about orthodontic procedures before treatment, and a written consent was signed and obtained by each patient, including the possibility to use their records. The final sample consisted of 35 white subjects, divided in two groups: 24 patients (13 males and 11 females) with a mean age of 12.9 ± 1.4 years (minimum 11 years and 3 months, maximum of 14 years and 3 months) were treated with the Segmented Pendulum and fixed appliances, and 11 patients (6 males and 5 females) with a mean age of 13.2 ± 1.2 years (minimum 11 years and 7 months, maximum 14 years and 6 months) were treated with the Quad Pendulum and fixed appliances. All patients presented erupted second molars: in the SP group, the first and second molars have been sequentially distalized (the second molar followed by the first molar); in the QP group, the first and second molars have been simultaneously distalized. The initial traits of the subjects in both groups were considered comparable (Table 1). The average amount of class II molar relationship was 2.6 mm in the SP and 2.3 mm in the QP, with a mean overjet of 4.9 and 3.8 mm, respectively, at the beginning of treatment. Three serial cephalograms for all patients were available at three observation times: before treatment (T1), after distalization (T2), and after orthodontic fixed appliance therapy (T3). Demographics of observation periods and observation interval are reported in Table 2.

**Table 1:** **Sample selection and exclusion criteria and their cephalometric values for Segmented Pendulum and Quad Pendulum**

	**Segmented pendulum group** ***n*** **= 24**		**Quad pendulum group** ***n*** **= 11**	
	**Mean**	**SD**	**Mean**	**SD**
Sagittal skeletal				
Maxillary position S-N-A	83.4°	0.9°	82.9°	0.8°
Mandibular position S-N-Pg	79.3°	1.1°	77.9°	1.3°
Sagittal jaw relation A-N-Pg	4.1°	1.1°	4.3°	1.0°
Vertical skeletal				
Maxillary inclination S-N/ANS-PNS	7.9°	0.6°	8.5°	0.7°
Vertical jaw relation ANS-PNS/Go-Gn	26.3°	2.5°	26.6°	2.2°
Mandibular inclination S-N/Go-Gn	34.2°	3.6°	35.1°	2.3°
Dento-basal				
Maxillary incisor inclination 1: ANS-PNS	113.2°	7.1°	115.9°	5.3°
Mandibular incisor inclination 1: Go-Gn	93.5°	6.3°	92.3°	4.9°
Mandibular incisor compensation 1: A-Pg (mm)	2.0	0.9	2.9	1.1
Dental relations				
Overjet	4.9	1.3	3.8	1.2
Overbite	2.7	1.1	3.3	0.9
Interincisal angle (1/1)	127.1°	6.3°	124.3°	4.6°
Molar relationship (mm)	2.6	0.6	2.3	0.5

Mann-Whitney *U* test.

**Table 2:** **Comparison of starting forms**

**Observational period/interval**	**Segmented pendulum group** ***n*** **= 24**				**Quad pendulum group** ***n*** **= 11**			
	**Mean**	**SD**	**Min**	**Max**	**Mean**	**SD**	**Min**	**Max**
T1	12 y 9 m	1 y 4 m	11 y 3 m	14 y 3 m	13 y 2 m	1 y 2 m	11 y 7 m	14 y 6 m
T2	13 y 7 m	1 y 3 m	12 y 3 m	15 y 3 m	14 y 3 m	9 m	11 y 10 m	16 y 4 m
T3	15 y 3 m	1 y 4 m	13 y 6 m	16 y 9 m	15 y 10 m	1 y 4 m	13 y 9 m	17 y 8 m
T1 to T2	10 m	2 m	7 m	12 m	11 m	1 m	7 m	12 m
T2 to T3	20 m	4 m	17 m	26 m	21 m	3 m	16 m	25 m
T1 to T3	30 m	3 m	25 m	36 m	32 m	4 m	23 m	37 m

y, years; m, months.

### Ethical approval

Ethical approval was not deemed necessary for this study. All patients were treated in a private practice where asking for radiographs at T1-T2-T3 is considered normal part of the orthodontic treatment; moreover, all patients were treated using a “conventional protocol” and any experimental procedure was used.

### Clinical management

All patients underwent maxillary molar distalization therapy with two different modified Pendulum appliances followed by fixed appliances. The two types of Pendulum appliances used in this study represented modifications [21] of the standard Pendulum of Hilgers [22]. The Segmented Pendulum is composed of two TMA springs and two distal screws (one for each side), whereas the Quad Pendulum is composed of four TMA springs (two for each side).

### Segmented Pendulum

The Segmented Pendulum allows sequential distalization of the second molar followed by the first molar. The appliance includes two distal screws (one for each side) dividing the Nance button into two sections. The anterior part provides anchorage, while the posterior includes distal screw and TMA springs, which represent the active elements of the appliance. Differently to the Pendulum-K described by Kinzinger et al. [14], the TMA springs are not activated by opening the distal screw. Distal screw is already opened at the beginning of treatment in order to get TMA wires close to the lingual sheath of the second molar (Figure 1a). The TMA springs are then activated intraorally by the clinician (300 g for each side) once or twice during the procedure adding uprighting bends to the end of the TMA wire in order to prevent excessive molar tipping [23]. Initially, the first molars are excluded from the anchorage unit, allowing them to partially follow the distal movement of the second molars. Once a ‘super class I’ molar relationship is achieved at the second molar (about 4 to 5 months), the distal screws are completely closed and the TMA springs disengaged from the second molar and inserted into the lingual sheaths of the first molar (Figure 1b).

Henceforth, the appliance is left ‘*in situ*’ until a ‘super class I’ molar relationship at the first molar is achieved. The mean treatment time for distalizing maxillary molars was 10 ± 2 months.

### Quad Pendulum

The Quad Pendulum allows simultaneous distalization of the second and first molars. The appliance is composed of a Nance button which provides anchorage and four TMA springs (two for each side) inserted into the lingual sheaths of the second and first molars at once. Differently to the Segmented Pendulum, distal screws are not present (Figure 2a). As recommended by Byloff and Darendeliler [23], uprighting bends were added to the end of the TMA wires to prevent excessive molar tipping and allow bodily distalization. The TMA springs are activated intraorally by the clinician (300 g for each molar) once or twice during the procedure, and the appliance is left ‘*in situ*’ until a ‘super class I’ molar relationship is achieved both at the first and second molars. The mean treatment time for distalizing maxillary molars was 11 ± 1 months.

After removal of the Segmented or Quad Pendulum, a Nance button on the first molar was placed and left passively for 4 to 5 months in order to retain the distalized first molars and allow a spontaneous premolar distal drifting. Subsequently, pre-adjusted fixed orthodontic appliances (Roth prescription, 0.022 in × 0.028 in) were placed. After conventional tooth leveling and aligning, the maxillary premolars and canine were individually retracted. Then, the Nance button was removed, and the anterior teeth were retracted using sliding mechanics; quarter-inch intermaxillary elastics in conjunction with fixed appliances were used as support in anchorage during incisor retraction.

The mean total treatment time was 2 years 6 ± 3 months for the Segmented Pendulum and 2 years 8 ± 4 months for the Quad Pendulum.

### Cephalometric analysis

**Figure 3:**
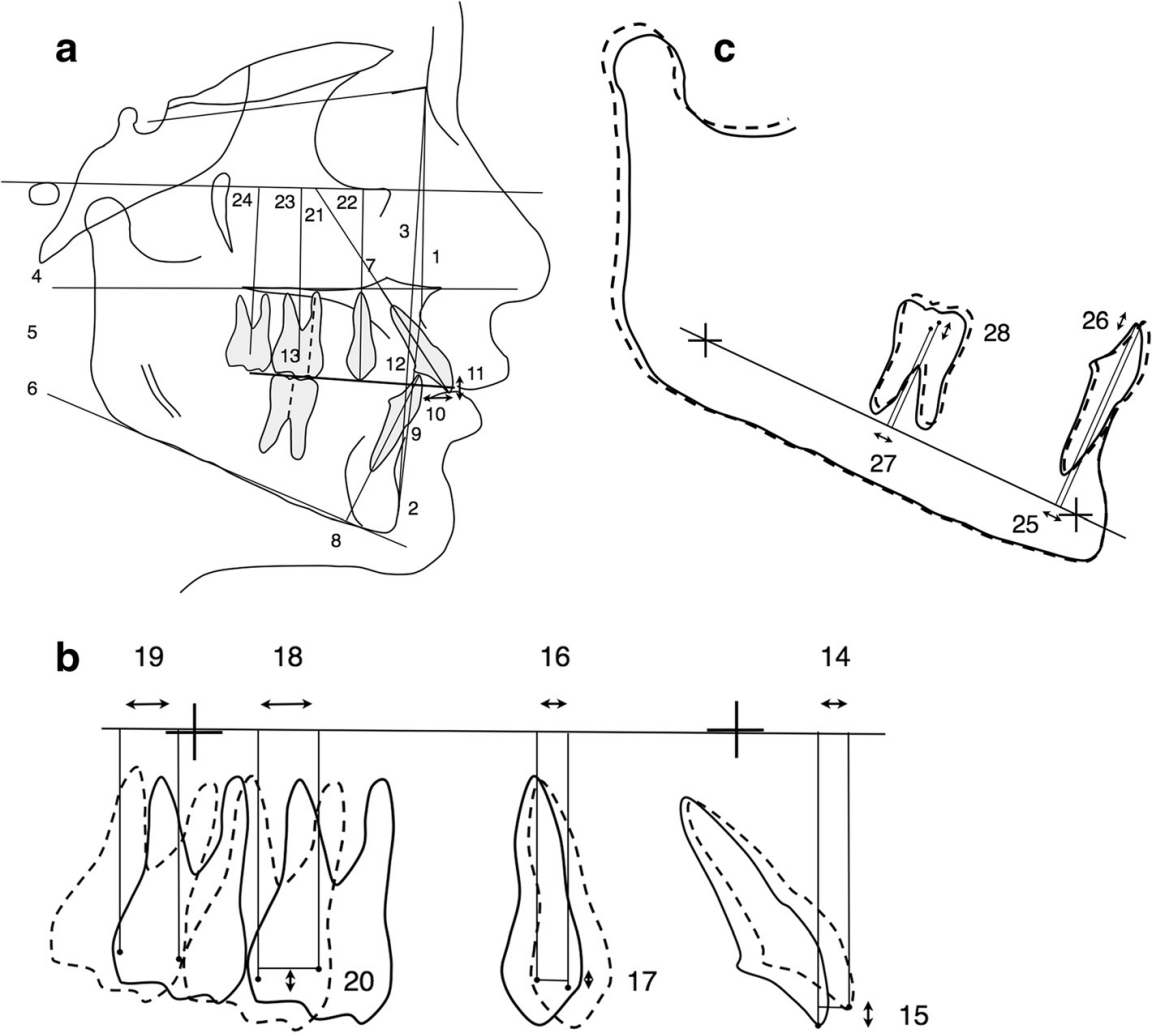
**Cephalometric landmarks and measurements (a, b, c).** (1) SNA, (2) SNPg, (3) ANPg, (4) SN/ANS-PNS, (5) ANS-PNS/Go-Gn, (6) SN/Go-Gn, (7) 1: ANS-PNS, (8) 1: Go-Gn, (9) 1/A-Pg, (10) overjet, (11) overbite, (12) interincisal angle, (13) molar relationship, (14) U1 horizontal, (15) U1 vertical (perpendicular to a line passing through the maxillary fiducial markers), (16) U4 horizontal, (17) U4 vertical, (18) U6 horizontal, (19) U7 horizontal, (20) U6 vertical, (21) U1 to FH, (22) U4 to FH, (23) U6 to FH, (24) U7 to FH, (25) L1 horizontal, (26) L1 vertical (perpendicular to a line passing through the mandibular fiducial markers), (27) L6 horizontal, and (28) L6 vertical.

Lateral cephalograms for each patient at T1, T2, and T3 in each treatment group were standardized as the same magnification factor (6% enlargement). Cephalograms were hand traced by a single investigator with verification of anatomic outlines, and landmark position was performed by a second investigator. In case of disagreement, the structures in question were retraced to the mutual satisfaction of both. In instances of bilateral structures (e.g., gonial angle and teeth), a single averaged tracing was made. The cephalometric analysis consisted of 32 landmarks, 13 angular measurements, and 15 linear measurements for each tracing; 4 fiducial markers were also placed in the maxilla and mandible [24] (Figure 3a,b,c). The 32 landmarks and the 4 fiducial markers were used for superimposition [25,26].

### Statistical analysis

Descriptive statistics was calculated for age, duration of treatment, and cephalometric measurements at T1 for the two groups. Significant between-group differences were tested with a Mann-Whitney *U* test for each cephalometric variable before treatment. No significant difference was found between the two groups. The mean differences and standard deviations were also calculated for the treatment changes between T1 to T2, T2 to T3, and T1 to T3. A Mann-Whitney *U* test was used to identify significant between-group differences for each cephalometric variable between T1 and T2, T2 and T3, and T1 and T3 with a statistical software package (MedCalc® Version 12.2.1, Mariakerke, Gent, Belgium). Statistical significance was tested at *p* < 0.05, *p* < 0.01, and *p* < 0.001.

### Method error

Fifteen randomly selected cephalograms were retraced by the same author after a period of 2 months. No significant mean differences between the two series of records were found by using paired *t* tests. Dahlberg's formula was used to establish the method error [27]. A range from 0.5 to 0.8 mm for linear measurements and 0.6° to 0.9° for angular measurements was found. Reliability coefficient (*r*) [28] ranged from 0.94 to 0.98 and from 0.92 to 0.97, respectively.

## Results

**Figure 4:**
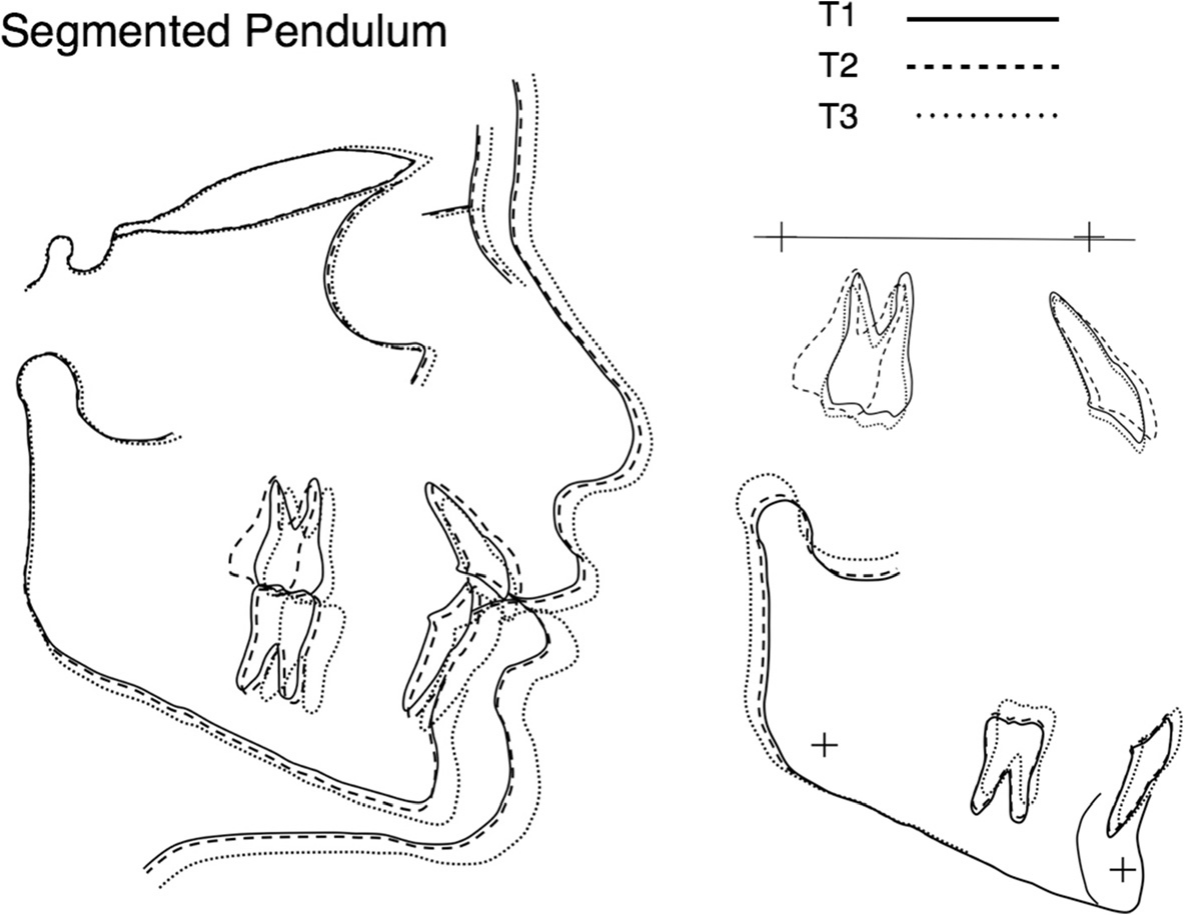
**Average craniofacial forms for Segmented Pendulum group at T1, T2, and T3.** Cranial base (left), maxillary (top right), and mandibular (lower right) superimpositions.

**Figure 5:**
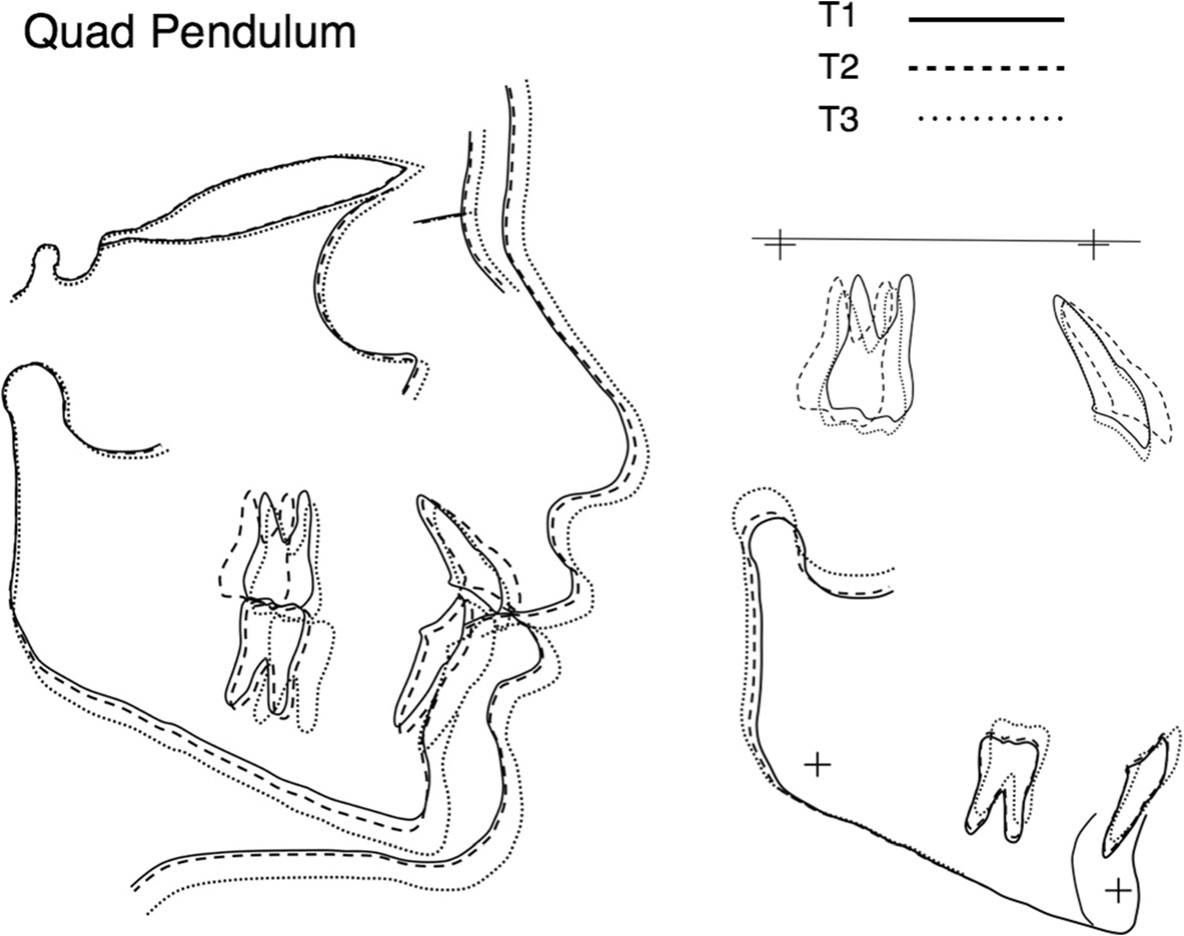
**Average craniofacial forms for Quad Pendulum group at T1, T2, and T3.** Cranial base (left), maxillary (top right), and mandibular (lower right) superimpositions.

The mean, standard deviation, and statistical significance of the dento-skeletal changes relative to T1 to T2, T2 to T3, and T1 to T3 are summarized in Table 3. Average craniofacial forms for both groups at the three observation times, and their superimpositions are shown in Figures 4 and 5.

**Table 3:** **Demographics of the observation periods and observation intervals**

	**T1 to T2**					**T2 to T3**					**T1 to T3**				
	**Segmented pendulum group**		**Quad pendulum group**			**Segmented pendulum group**		**Quad pendulum group**			**Segmented pendulum group**		**Quad pendulum group**		
**Cephalometric measures**	**Mean**	**SD**	**Mean**	**SD**	***p*** **value**	**Mean**	**SD**	**Mean**	**SD**	***p*** **value**	**Mean**	**SD**	**Mean**	**SD**	***p*** **value**
Sagittal skeletal relations															
Maxillary position S-N-A	0.30	0.80	0.40	1.10	0.32	0.60	0.60	0.70	0.40	0.14	0.90	0.70	1.10	0.80	0.36
Mandibular position S-N-Pg	−1.00	0.50	−1.80	0.90	0.18	3.50	0.90	3.00	1.06	0.45	2.50	1.10	1.20	0.80	0.61
Sagittal jaw relation A-N-Pg	1.00	0.60	1.20	0.40	0.35	−3.00	0.50	−3.00	0.30	0.57	−2.00	0.60	−1.80	0.40	0.26
Vertical skeletal relations															
Maxillary inclination S-N/ANS-PNS	0.30	0.60	0.40	0.20	0.12	0.30	0.60	0.40	0.50	0.16	0.60	0.60	0.80	0.40	0.31
Vertical jaw relation ANS-PNS/Go-Gn	0.40	0.60	0.60	0.50	0.63	0.40	0.40	0.60	0.50	0.14	0.80	0.50	1.20	0.50	0.0113*
Mandibular inclination S-N/Go-Gn	0.70	0.90	1.20	0.60	0.0038**	0.70	0.40	0.90	0.50	0.47	1.40	0.70	2.60	0.60	0.0026**
Dento-basal relations															
Maxillary incisor inclination 1: ANS-PNS	4.10	0.90	6.10	1.10	0.0019**	−5.20	1.10	−9.10	1.00	<0,001***	−1.10	1.00	−3.00	1.10	0.0316*
Mandibular incisor inclination 1: Go-Gn	0.20	0.60	0.10	0.80	0.59	0.70	0.60	1.10	0.90	0.23	0.90	0.60	1.20	0.90	0.12
Mandibular incisor compensation 1/A-Pg (mm)	0.10	0.60	0.10	1.00	0.23	0.40	0.50	0.70	0.60	0.22	0.50	0.60	0.80	0.80	0.74
Dental relations															
Overjet (mm)	1.90	0.70	3.90	0.50	<0.001***	−4.80	0.80	−5.20	0.40	0.0153**	−2.90	0.80	−1.30	0.50	0.41
Overbite (mm)	−0.20	0.60	−2.10	0.60	<0.001***	−0.50	0.70	0.80	0.40	0.0471*	−0.70	0.70	−1.30	0.50	0.0185*
Interincisal angle (1/1)	−4.10	1.10	−5.10	1.20	0.18	7.30	1.10	8.10	0.90	0.51	3.20	1.10	3.00	1.10	0.16
Maxillary dentoalveolar															
Molar relationship (mm)	−4.00	1.00	−3.50	0.90	0.48	0.70	0.80	1.00	0.70	0.31	−3.30	0.90	−2.50	0.80	0.16
U1 horizontal (mm)	1.70	0.70	2.10	0.90	0.67	−2.60	0.40	−3.40	0.40	0.0035**	−0.90	0.60	−1.30	0.70	0.28
U1 vertical (mm)	−0.4	1.00	−1.60	1.20	0.11	1.00	0.70	1.50	0.80	0.15	1.00	0.90	−0.10	1.00	0.0321*
U4 horizontal (mm)	1.50	1.20	1.80	0.90	0.16	−2.70	0.30	−2.90	0.20	0.15	−1.20	0.80	−1.10	0.60	0.36
U4 vertical (mm)	0.30	0.20	0.80	0.90	0.38	0.10	0.40	0.20	0.90	0.44	0.40	0.30	1.00	0.90	0.52
U6 horizontal (mm)	−4.00	0.90	−3.50	0.70	0.26	2.20	0.60	2.00	0.70	0.49	−1.80	0.80	−1.50	0.70	0.17
U7 horizontal (mm)	−3.80	1.20	−3.20	0.90	0.42	1.90	0.40	1.90	0.60	0.58	−1.90	0.80	−1.30	0.70	0.16
U6 vertical (mm)	0.20	0.20	1.10	0.90	<0.001***	0.80	0.60	0.70	0.40	0.37	1.00	0.40	1.80	0.70	<0.001***
U1 to FH (deg)	3.80	0.90	6.60	1.20	0.0019**	−4.90	0.70	−9.10	0.90	<0.001***	−1.10	0.80	−2.50	1.10	<0.001***
U4 to FH (deg)	1.60	0.90	2.00	0.70	0.13	−0.90	1.10	−1.30	0.70	0.12	0.70	1.00	0.70	0.70	0.27
U6 to FH (deg)	−9.60	1.60	−4.60	1.10	<0.001***	10.10	1.10	5.80	0.90	<0.001***	0.50	1.40	1.20	1.00	0.17
U7 to FH (deg)	−14.60	4.60	−11.60	5.10	0.0454*	13.00	3.40	11.30	4.20	0.53	−1.60	1.40	−0.30	1.00	0.31
Mandibular dentoalveolar															
L1 horizontal (mm)	0.20	0.30	0.10	0.40	0.46	0.90	0.70	1.30	0.50	0.0237*	1.10	0.50	1.40	0.50	0.14
L1 vertical (mm)	0.30	0.20	0.60	0.30	0.54	0.40	0.40	0.50	0.60	0.66	0.70	0.30	1.10	0.50	0.26
L6 horizontal (mm)	0.10	0.40	0.20	0.40	0.67	0.60	0.40	0.90	0.40	0.13	0.70	0.40	1.10	0.40	0.32
L6 vertical (mm)	0.30	0.40	0.30	0.50	0.17	1.30	0.60	1.10	0.70	0.12	1.60	0.50	1.40	0.60	0.63

Mann-Whitney *U* test. *p < 0.05; **p < 0.01; ***p < 0.001.

### Pre-treatment to post-distalization (T1 to T2)

No significant sagittal skeletal change was detected between the two groups during the distalization phase, whereas skeletal vertical change revealed a greater mandibular inclination in the QP group (S-N/Go-Gn = 1.2° ± 0.6°) than in the SP group (0.7° ± 0.9°) (*p* < 0.01).

Maxillary molars showed a mean distal movement of 4.0 ± 0.9 mm in SP and 3.5 ± 0.7 mm in QP, but these changes were not statistically significant. However, maxillary first molars showed greater distal tipping (U6 to FH = −9.6° ± 1.6° vs. −4.6° ± 1.1°; *p* < 0.001) and less extrusion (U6-vertical = 0.2 ± 0.2 mm vs. 1.1 ± 0.9 mm; *p* < 0.001) in the SP than in the QP. Mean treatment time for distalization was 10 ± 2 months in the SP and 11 ± 1 months in the QP.

No significant difference was found in the anchorage loss: the first premolar did not show any statistically significant difference both in the mesial and vertical movement between the two groups. However, the maxillary incisors in the QP showed a greater proclination (6.1° ± 1.1°) in comparison with the SP (4.1° ± 0.9°) (*p* < 0.01), as well as the overjet which increased 3.9 ± 0.5 mm vs. 1.9 ± 0.7 mm, respectively, (*p* < 0.001). Overbite decreased more in the QP (−2.1 ± 0.6 mm) than in the SP (−0.2 ± 0.6 mm) (*p* < 0.001).

### Post-distalization to the end of orthodontic treatment (T2 to T3)

No statistically significant sagittal and vertical skeletal change was found between the two groups. The maxillary first molars showed a mesial movement in both groups, 2.2 ± 0.6 mm in the SP and 2.0 ± 0.7 mm in the QP, and a slight extrusion (0.8 ± 0.6 mm vs. 0.7 ± 0.4 mm, respectively), but no statistical difference was detected between the two groups; significant difference was found in molar tipping, which was completely corrected both in the SP (U6 to FH = 10.1° ± 1.1°) and in the QP (5.8° ± 0.9°) (*p* < 0.001).

The first premolar distalized 2.7 ± 0.3 mm in SP and 2.9 ± 0.2 mm in QP, but these changes were not statistically significant. Maxillary incisors retroclined more in the QP (9.1° ± 0.9°) than in the SP (4.9° ± 0,7°) (*p* < 0.001), as well as the overjet, which decreased to 5.2 ± 0.4 mm and 4.8 ± 0.8 mm, respectively (*p* < 0.05). Mandibular molars showed mesial movement and extrusion in both groups, but these changes were not statistically significant.

### Overall treatment effect (T1 to T3)

Sagittal skeletal relation did not show any significant change between the two groups. On the contrary, vertical facial dimension increased more in the QP (2.6° ± 0.6°) than in the SP (1.4° ± 0.7°) (*p* < 0.01). Maxillary molars were in a more distal position than they were before treatment in both groups (−1.8 ± 0.8 mm in the SP and −1.5 ± 0.7 mm in the QP), and molar tipping did not show any significant difference between the groups (0.5° ± 1.4° in the SP and 1.2° ± 1.0° in the QP). However, QP showed greater molar extrusion (1.8 ± 0.7 mm) than SP (1.0 ± 0.4 mm) at the end of treatment (*p* < 0.001). Class II molar relationship was corrected by distal molar movement and a concomitant mandibular mesial molar movement and mandibular advancement (S-N-Pg = 2.5° ± 1.1° in the SP and 1.2° ± 0.8° in the QP), but these changes did not show any significant difference between the groups. The overbite decreased more in the QP (−1.3 ± 0.5 mm) than in the SP (−0.7 ± 0.7 mm) (*p* < 0.05), whereas overjet did not show any significant difference between the groups.

## Discussion

Several studies [15,23,29,30] evaluated the efficacy of the Pendulum appliance, reporting data relative to dentoalveolar and skeletal changes, but few studies [7,14,31] described molar distalization in the presence of fully erupted second molar. Segmented and Quad Pendulums were previously described by Kinzinger et al. in a case report study [21] and differed from most distalizing devices since TMA wires acted directly on second molars, without being distalized by a force applied on the first molar. Appliance design was based on the concepts of Gianelly [17] and Jeckel and Rakosi [18]; according to them, when the second molars have erupted, the distalization of the molars should be done in stages, first, the second molars and then the first molars.

No significant difference was found in molar distalization between the two appliances. The second molar distalized 3.8 mm in the SP and 3.2 mm in the QP, and the first molar distalized 4 and 3.5 mm, respectively, between T1 and T2. Kinzinger et al. [14] and Bussick and McNamara [15] demonstrated that the amount of molar distalization might not be influenced by the presence of erupted second molar (3.1 vs. 3.2 mm, respectively, using Pendulum-K; 5.7 vs. 5.6 mm using Pendulum appliance), as well as Fontana et al. [32] who demonstrated the same results in adult patients. These findings agreed with those of Ghosh and Nanda [13] and Muse et al. [33], who stated that maxillary first molar distalization can be accomplished before or after the eruption of the second molars with no appreciable or significant differences in outcomes.

Significant differences were found in distal tipping. The first molar tipped a mean of 9.6° in the SP and 4.6° in the QP during the distalization phase, suggesting that concomitant distal movement of the first and second molars (QP) may result in less tipping [14]. Tipping resulting in a sequential distalization (SP) may be explained by the inherent feature of the Pendulum itself [12], despite uprighting bend on the TMA wires [23]. In support of this hypothesis, Bussick and McNamara [15] reported that distal tipping occurred in the absence of the second molar (11.7°) and in presence of erupted second molar (9.8°) using Pendulum appliance with no significant difference. Kinzinger et al. [14], as well as Karlsson and Bondemark [7], in fact showed less tipping using a modified Pendulum-K (0.9°) and compressed palatal coils (3°). However, both QP and SP showed marked distal tipping of the second molar between T1 and T2 (14.6° in the SP and 11.6° in the QP). This was well explained by Kinzinger et al. [14], who showed that the distal movement of the second molar in the presence of third molar's bud was accompanied by a marked distal tipping, according to the ‘fulcrum theory’. Most of the articles did not provide data relative to the second molar during the distalization phase, making difficult to compare our data with those from studies that use other appliances, in which the second molar was moved distally by a force acting on the first molar. However, during fixed appliance therapy (T2 to T3), the mesial movement of the maxillary molars was noted in both appliances, and distal tipping was completely corrected, but part of the distal movement (about 45%) was maintained at the end of the treatment (1.8 mm in the SP and 1.5 mm in the QP), confirming the findings previously reported in a long-term investigation using Pendulum appliance (57%) [26]. However, it can be noted that most of the class II correction can be achieved by a favorable mandibular growth in both groups (Figures 4 and 5). Despite the greater amount of molar tipping produced by the SP than the QP, total treatment time was similar in both groups (30 and 32 months, respectively) which may indicate that anchorage loss during distalization would more influence treatment time than molar tipping.

Mesial movement of the premolars and labial tipping of the incisors generally occur as unavoidable negative effects [10]. The first premolar showed a mean mesial movement of 1.5 mm in the SP and 1.8 mm in the QP, the maxillary incisor proclined 3.8° and 6.6°, and overjet increased 1.9 and 3.9 mm, respectively, indicating that sequential distalization could determine less anchorage loss. Similar findings were reported in previous studies, which showed maxillary incisor proclination of 5.5° using Pendulum-K [14], 3.9° using compressed coils [7], and 4° using Pendulum appliance [15] in the presence of the second molar with no substantial difference in the absence of the second molar (less than 2°). However, this could be considered a temporary effect; in fact, maxillary incisor and overjet were completely corrected during fixed appliance therapy (T2 to T3) using both appliances (−4.9° and −4.8 mm in the SP; −9.1° and −5.2 mm in the QP) using anchorage reinforcement, such as Nance button during retraction of premolar and canine and intermaxillary elastics during retraction of the incisor with sliding mechanics.

Significant differences were found in the vertical facial dimension. SP showed a mean increase in the SN/Go-Gn angle of 0.7° and QP of 1.2° during the distalization phase and a total increase of 1.4° and 2.6°, respectively, at the end of the overall orthodontic treatment (T1 to T3). Clockwise rotation of the mandible may occur as a consequence of the ‘wedge effect’ (which is related to the amount of molar distalization) and molar extrusion (11), which was greater in QP (1.1 mm) than in SP (0.2 mm) between T1 and T2. Bussick and McNamara [15] showed that molar extrusion was greater in presence of the second molar (0.4 vs. −0.5 mm), as well as the lower anterior facial height increased (2.7 vs. 1.5 mm), suggesting that the presence of the erupted second molar can be associated to a significant increase in vertical facial dimension and decrease in overbite. However, these findings were in disagreement with those of Kinzinger et al. [14] and Karlsson and Bondemark [7].

Most studies reported increase in vertical facial dimension during distalization phase (Byloff and Darendeliler [23] = +0.35°, Ghosh and Nanda [13] = +1.1°, Bussick and McNamara [15] = 1.2°). However, these studies did not consider data either at the end of fixed appliance therapy or long-term observation. In fact, other studies demonstrated that vertical facial dimension [25,34,35] increased during distalization, but decreased at the end of the orthodontic treatment. These findings were also confirmed by a long-term investigation [26], which reported 76 class II patients treated with Pendulum and fixed appliances describing that SN/Go-Gn angle increased 1.3° during the overall orthodontic treatment, but decreased 1.5° in the post-retention period (7 years later, age 22 years and 5 months). Therefore, although Quad Pendulum caused greater increase in the vertical dimension compared to the Segmented Pendulum, this phenomenon can be considered only a temporary effect after maxillary molar distalization, which can be partially or completely compensated by residual growth of the mandibular ramus after the completion of orthodontic treatment, leading to the return of the initial sagittal and vertical mandibular positions and maintenance of the mesofacial growth pattern throughout the treatment [26,35]. However, due to the limitations of a retrospective study, outcomes should be interpreted with caution, and further longitudinal long-term studies would be needed in order to confirm our findings and provide data using distalizing appliances anchored both on the first and second molars.

## Conclusions

– Based on the above results and discussion, the following conclusions were deduced:

– The hypothesis that Segmented and Quad Pendulums showed similar dental and skeletal changes has been rejected.

– SP and QP are equally effective to distalize the maxillary first and second molars; moreover, distalization phase and total treatment time were similar in both groups.

– However, SP showed greater molar distal tipping and less proclination of the maxillary incisor, whereas QP induced greater bodily distalization but more marked proclination of the incisors during the distalization phase.

– Moreover, QP showed more marked extrusion of the maxillary molar and increase in the vertical facial dimension, which is maintained at the end of comprehensive orthodontic treatment.
